# Unraveling Spermatogenesis in Molly Fish (*Poecilia sphenops*): An Integrative Study of Testicular Ultrastructure and Immunohistochemistry

**DOI:** 10.3390/vetsci12100930

**Published:** 2025-09-24

**Authors:** Doaa M. Mokhtar, Giacomo Zaccone, Marialuisa Aragona, Maria Cristina Guerrera, Rasha Alonizan, Manal T. Hussein

**Affiliations:** 1 Department of Cell and Tissues, Faculty of Veterinary Medicine, Assiut University, Assiut 71526, Egypt; manal.hussein@aun.edu.eg; 2 Department of Anatomy and Histology, School of Veterinary Medicine, Badr University in Assiut, Assiut 71526, Egypt; 3 Department of Veterinary Sciences, University of Messina, 98168 Messina, Italy; 4 Zebrafish Neuromorphology Lab, Department of Veterinary Sciences, University of Messina, Polo Universitario dell’ Annunziata, 98168 Messina, Italy; mlaragona@unime.it (M.A.); mguerrera@unime.it (M.C.G.); 5 Department of Zoology, College of Science, King Saud University, P.O. Box 2455, Riyadh 11451, Riyadh, Saudi Arabia; ralonezan@ksu.edu.sa

**Keywords:** calretinin, vimentin, Sertoli cells, Leydig cells, spermatozoa

## Abstract

**Simple Summary:**

This study provides a detailed analysis of the testis structure in *Poecilia sphenops*, highlighting its restricted lobular type and synchronized cystic spermatogenesis. Specialized sperm duct features and secretory activity support internal fertilization. Vimentin and calretinin expression confirmed the roles of Sertoli and Leydig cells, while TEM revealed ultrastructural adaptations for sperm development and delivery. These findings enhance the understanding of reproductive specialization in viviparous teleosts.

**Abstract:**

Spermatogenesis in teleosts is essential for reproductive function; however, it varies considerably among species. The testis of the viviparous molly fish (*Poecilia sphenops*) was examined using both ultrastructural and immunohistochemical methods. The testis displays a restricted lobular type, where germ cells develop synchronously within Sertoli cell-forming cysts. Transmission electron microscopy (TEM) revealed all stages of spermatogenesis. Mature sperm are at the apex of the cysts and migrate toward the sperm ducts. Sperm duct epithelium is lined by cuboidal cells joined by tight junctions, with apical cilia and desmosomal complexes contributing to transport and structural integrity. The sperm ducts showed strong Periodic Acid–Schiff (PAS)-positive expression among negative stained spermatocysts. Centrally, a cavity serves as a storage area for spermatozoa that are organized into unencapsulated bundles known as spermatozeugmata. Sertoli cells exhibited extended cytoplasmic processes that supported developing germ cells, whereas Leydig cells occupied the interstitial tissue, contributing to hormonal regulation. Immunohistochemical labeling demonstrated strong vimentin expression in Sertoli cells and telocytes, indicating their mesenchymal origin and structural role. Calretinin expression was confined to Leydig cells and certain ductal epithelial cells, supporting its use as a marker for steroidogenic and secretory functions. These findings provide new insights into the testicular specialization of *P. sphenops*, highlighting key somatic–germ cell interactions, ductal adaptations, and marker expression patterns that underlie male reproductive success in viviparous fish.

## 1. Introduction

Spermatogenesis in teleost fish exhibits remarkable diversity in both morphology and regulation, reflecting the wide range of reproductive strategies among species [1]. Spermatogenesis is a multistage, intricate process that is coordinated by both paracrine and endocrine cues. As mitosis, meiosis, and spermiogenesis advance, these signals are necessary to sustain the formation of four or five germ cell layers within each seminiferous tubule [2].

Viviparity is a rare trait in teleosts that has independently evolved at least 13 times and is found in only 2% of extant species [3]. However, viviparous fish, such as Poeciliidae, have evolved intricate testicular adaptations and internal fertilization to ensure the successful transfer of spermatozoa to the female reproductive system. The embryo develops in the ovary or uterus with nutrients supplied by the mother, whereas oviparous fish release eggs and fertilize externally [4]. These variations are reflected in the testis structure, the development of germ cells, the intricacy of the duct system, and the function of somatic cells [5].

Two main testicular types, lobular and anastomosing tubular testes, have been identified in teleosts. Depending on how spermatogonia are distributed and arranged, lobular testes can be arranged in two different ways [6]. Most oviparous teleosts have the unrestricted spermatogonial testis type in which spermatogonia are organized along the lengths of lobules. The restricted spermatogonial testis type involves spermatogonia that are confined to the periphery of the lobules and give rise to cysts formed by Sertoli cells. This restricted type is a feature of viviparous taxa, including the Goodeidae, Anablepidae, and Poeciliidae [7,8]. Comparative research indicates that this cystic spermatogenesis is shared by several Atherinomorpha lineages, including viviparous species (such as *Poecilia reticulata* and *Xiphophorus hellerii*) and ovoviviparous species. However, it has undergone structural modifications to facilitate internal insemination [5,9].

Sertoli and Leydig cells, somatic cells within the testis, also show species-specific adaptations. In viviparous fish, Sertoli cells completely enclose germ cell cysts and are responsible for nourishing, protecting, and synchronizing spermatogenesis [10]. Leydig cells, which are located in the interstitial tissue, produce androgens that regulate both spermatogenesis and secondary sexual traits [11]. Immunohistochemical markers such as vimentin, typically expressed in Sertoli cells, and calretinin, found in steroidogenic Leydig cells of several vertebrates, are valuable tools for differentiating somatic cell populations in the testis [12,13]. However, their roles and distribution patterns in teleost gonads remain poorly characterized, warranting further investigation.

Given the importance of *Poecilia sphenops* as a model for viviparous teleost reproduction and its frequent use in recent studies [14,15,16,17], a comprehensive analysis of its testicular structure is warranted. This study integrates light microscopy, TEM, PAS histochemistry, and immunohistochemistry to describe in detail the testicular organization, spermatogenic progression, sperm duct anatomy, and somatic cell marker expression in *P. sphenops*. Through comparison with related species, this work contributes to a better understanding of the evolutionary specialization of male reproductive systems in viviparous teleosts.

## 2. Materials and Methods

### 2.1. Sample Collection

This study was authorized by Assiut University′s Ethics Committee in Egypt (ethical number: aun/vet/4/0015). A total of 20 adult male molly fish (*Poecilia sphenops*) were used in this study (Figure S1). The specimens were randomly obtained from a single ornamental fish supplier during the summer season (June to August) to reduce potential variability due to genetic or environmental factors. All fish were of similar age (approximately 5–6 months), with a uniform average body weight of 9.20 ± 1.20 g and a standard length of 3.50 ± 0.4 cm, indicating that they were at comparable developmental stages. Prior to sample collection, the fish were acclimatized for two weeks under controlled laboratory conditions, with water temperature maintained at 26 ± 1 °C and a 12:12 h light–dark cycle to ensure environmental consistency. Euthanasia was performed using an overdose of tricaine methanesulfonate (MS-222, 3%) in compliance with the ethical guidelines for animal research [18].

### 2.2. Histological and Histochemical Analyses

Ten whole fish were promptly fixed post-mortem in Bouin’s solution for 22 h. After fixation, the specimens were dehydrated through a graded ethanol series and cleared using methyl benzoate. The tissues were then embedded in paraffin wax. Serial sections, each 5 μm thick, were prepared. Sections were stained using a combination of Periodic Acid–Schiff (PAS), Alcian Blue (AB), and Hematoxylin (HX) to visualize different histochemical features. PAS staining was performed to detect glycogen and neutral glycoproteins. Alcian Blue selectively highlighted acidic mucopolysaccharides and glycosaminoglycans. Hematoxylin provided nuclear counterstaining, allowing clear visualization of tissue architecture and cell morphology [19].

For each specimen, serial sagittal and transverse sections were prepared, and at least five representative sections per testis were examined for histological and immunohistochemical analysis. The structural characteristics of the different types of testicular cells were established based on previous studies [5,6,9].

### 2.3. Semithin Sections and TEM

The testes from ten fish specimens were carefully dissected and fixed in a 2.5% paraformaldehyde–glutaraldehyde mixture overnight. After rinsing in 0.1 M phosphate buffer, the samples were post-fixed with 1% osmium tetroxide prepared in 0.1 M sodium cacodylate buffer (pH 7.3). The tissues were then dehydrated through a graded ethanol series, followed by the addition of propylene oxide, and embedded in Araldite resin. Semithin sections (1 µm) were stained with toluidine blue and examined under a Letiz Dialux 20 microscope (Ernst Leitz Wetzlar GmbH in Wetzlar, Germany) and a Canon digital camera (Candison Powershot A95) (Canon Inc., Tokyo, Japan). Ultrathin sections (~70 nm) were prepared using an Ultrotome V (LKB Bromma, Stockholm, Sweden), stained with uranyl acetate and lead citrate, and subsequently examined using a JEOL-100CX II transmission electron microscope (JEOL Ltd, Tokyo, Japan).

### 2.4. Localization of Calretinin and Vimentin Using Indirect Peroxidase Immunohistochemistry Staining

Some serial sections were deparaffinized and rehydrated, washed in Phosphate-Buffered Saline (PBS) (0.1 M; pH = 7.4), and incubated in 0.3% H_2_O_2_ (PBS) solution for 3 min to prevent endogenous peroxidase activity; then, fetal bovine serum (F7524 Sigma-Aldrich) was added to rinsed sections. Sections were incubated overnight at 4 °C in a humid chamber with anti-Calretinin and anti-Vimentin antibodies (Table 1). After rinsing in PBS, the sections were incubated for 1.5 h at room temperature with anti-goat IgG-peroxidase and anti-mouse IgG-peroxidase conjugates (Table 1). The immunoreaction was visualized using 3-30-diaminobenzidine as a chromogen (according to the manufacturer’s instructions, Sigma-Aldrich, D5905). After rinsing in freshwater, the sections were dehydrated, mounted, and examined using the Leica Application Suite LAS V4.7 under a Leica DMRB light microscope equipped with a Leica DFC7000 T camera (Leica Microsystems GmbH, Wetzlar, Germany). The primary antibodies against vimentin and calretinin used in this study were selected based on previous validation in teleost fish tissues [20,21]. To confirm specificity in *Poecilia sphenops*, negative control sections were processed identically but with omission of the primary antibody, which resulted in no detectable signal. These controls confirmed the reliability of the observed immunostaining patterns (Figure S2).

### 2.5. Digitally Colored TEM Images

To enhance the visual contrast between numerous structures on a single electron micrograph, we artificially colored some features to make them more noticeable to readers. Hand-coloring of each element was performed with care using Adobe Photoshop version 6. All original, non-color-adjusted images are included in Figures S3–S6.

### 2.6. Quantification of Spermatogenic Stages and Morphometric Analysis of Ductal Epithelium

For each specimen, five representative lobules per section were analyzed in three different sections. Spermatogenic cysts were classified as spermatogonia, primary spermatocytes, secondary spermatocytes, spermatids, or spermatozoa based on morphological criteria. The number of cysts in each category was counted using ImageJ software (v. 1.52t), and the percentage of each stage was calculated relative to the total number of cysts. Data are presented as the mean ± SD for all specimens. In addition, the heights of the efferent and sperm duct epithelium were measured in sections stained with toluidine blue, and measurements were taken from the basement membrane to the luminal surface at ten random points per duct. At least three ducts per specimen were analyzed, and mean values were calculated for each fish before determining the overall mean ± SD. Statistical analysis was performed using one-way ANOVA followed by Tukey’s post hoc test to determine differences among stages, with significance set at *p* < 0.05. Analyses were performed using “GraphPad Software” (CA, USA, Version 6.05).

## 3. Results

### 3.1. Light Microscopy

**Figure 1:**
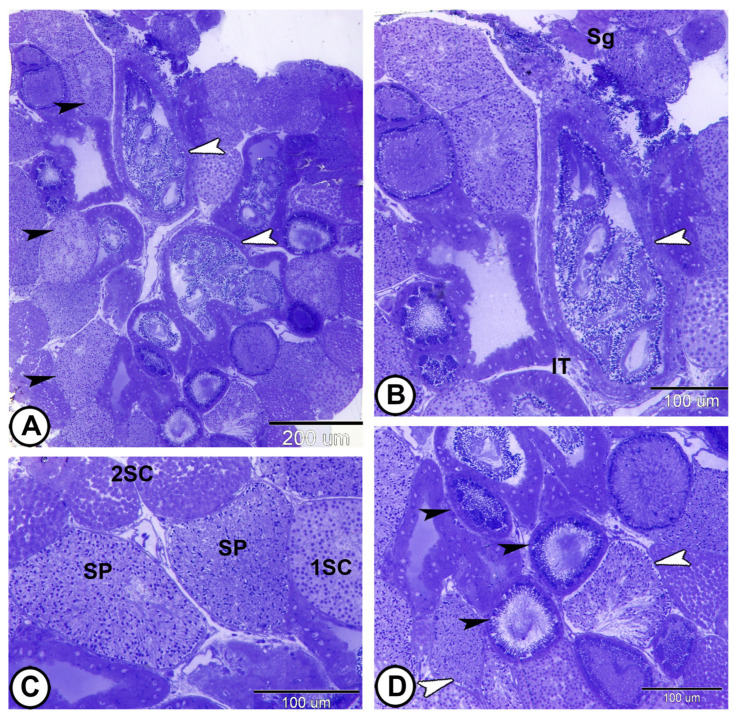
**Semithin sections stained with toluidine blue showing the general morphology of the testis of molly fish.** (**A**,**B**) The testis is a lobular type, containing many cysts (black arrowheads). Spermatozoa are stored in a central cavity (white arrowheads). Note the peripheral spermatogonia (Sg) and interstitial tissue (IT). (**C**) Spermatogenesis occurs synchronously within these cysts. The cysts enclose germ cells [primary spermatocytes (1SC), secondary spermatocytes (2SC), and spermatozoa (SP)] at the same stages of development. (**D**) As spermatogenesis progresses, these cysts (white arrowheads) move toward the center of the testis and eventually to the efferent ducts (black arrowheads). (Scale bars: 200 μm in (**A**); 100 μm in (**B**–**D**)).

Molly fish had a restricted spermatogonial lobular type of testis, which was distinguished by spermatogonia that were restricted to the peripheral. The testicular parenchyma was organized into lobules packed with cysts and interstitial tissue. A large cavity was located at the center of the testis, where the spermatozoa are stored (Figure 1A,B). Each cyst enclosed germ cells at the same developmental stage (Figure 1C). As spermatogenesis progresses, these cysts move toward the testis center and eventually to the efferent ducts (Figure 1D).

**Figure 2:**
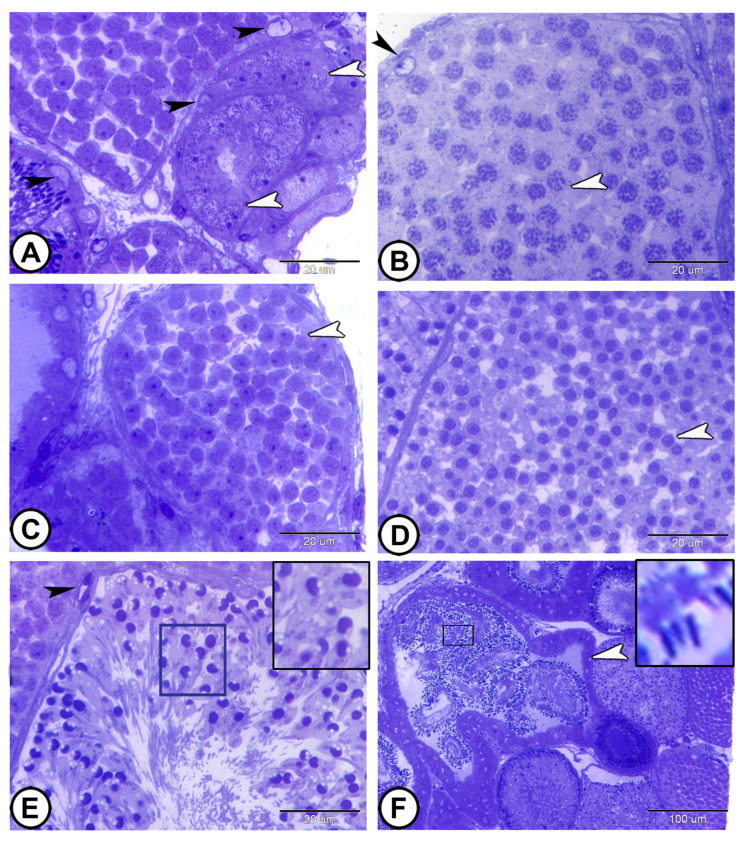
**Semithin sections stained with toluidine blue showing the stages of spermatogenesis.** (**A**) The spermatogonia cysts (white arrowheads). The cysts are formed by Sertoli cells (black arrowhead). (**B**) Primary spermatocytes with filamentous chromatin (white arrowhead). Note the presence of Sertoli cells (black arrowhead). (**C**) Secondary spermatocytes with thinner filamentous chromosomes (white arrowhead). (**D**) Spermatids (white arrowhead). (**E**) Horseshoe-shaped spermatid cysts (boxed areas) surrounded by Sertoli cells (black arrowhead). (**F**) Sperms migrating to the efferent duct (white arrowhead). Note the elongated nuclei of sperms (boxed areas). (Scale bar: 20 μm in (**A**–**F**)).

The process of spermatogenesis took place inside the cysts, with each cyst′s germ cells developing synchronously. The germ cells were grouped in spermatocysts and surrounded by Sertoli cell processes (Figure 2A,B). Spermatogonia cysts are the structural unit of spermatogenesis. They are usually demonstrated peripherally and are characterized by a large nucleus containing a distinct nucleolus (Figure 2A). Spermatogonia were divided mitotically to produce primary spermatocytes that were larger but smaller than spermatogonia. Primary spermatocytes were spherical cells with filamentous chromatin in the nucleus, a characteristic feature of the pachytene stage (Figure 2B).

Secondary spermatocytes were smaller than primary spermatocytes and were produced by the first meiotic divisions. These cells had a spherical nucleus with thinner, filamentous chromosomes. Because secondary spermatocytes enter the second phase of meiosis following a short interphase to generate spermatids, they were smaller in number than primary spermatocytes (Figure 2C). By the second meiotic division, spermatids were produced (Figure 2D). All sperms within cysts mature at the same time. Early spermatids retained the spherical shape of secondary spermatocytes; their nuclei were highly condensed and developed the shape of a horseshoe (Figure 2D,E). In late spermatids, the flagellum then began to grow, and the nucleus elongated. The development of a nuclear fossa at the base of the nucleus, which gave it an arrowhead shape, was indicative of nuclear elongation (Figure 2F). Spermatozeugmata, or unencapsulated sperm bundles, were the form in which the spermatozoa were produced. In the latter, the spermatozoa tails were spirally oriented in the center, while their heads, which were connected to the Sertoli cells lining the cysts, pointed outward (Figure 2E). Following that, the sperm migrated to the efferent duct (Figure 2F).

**Figure 3:**
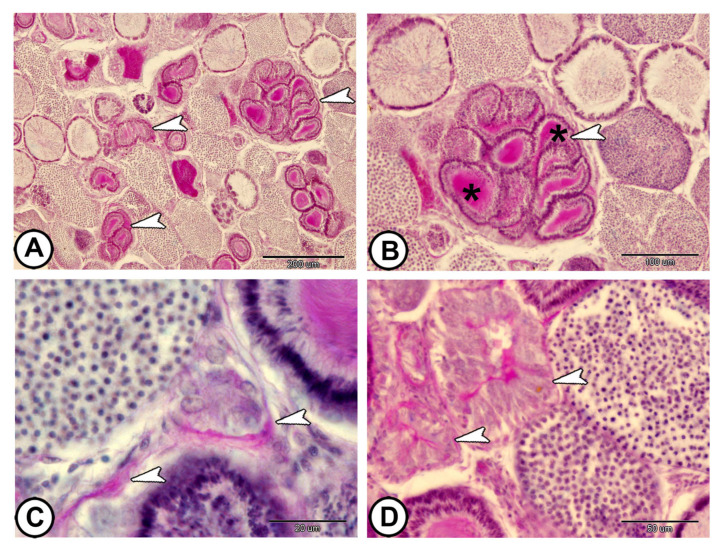
**PAS/AB/HX staining of the testis.** (**A**,**B**) The sperm ducts (arrowheads) showed strong positive expression. Note that the sperm ducts contain a strong PAS–mucoprotein complex (asterisks). (**C**) The cytoplasm of Sertoli cells shows strong PAS reaction (arrowheads). (**D**) Spermatocytes exhibiting positive PAS/AB reaction (arrowheads). (Scale bars: 200 μm in (**A**); 100 μm in (**B**); 20 μm in (**C**); and 50 μm in (**D**)).

The basement membranes of the cysts were clearly visible when stained with PAS. The PAS–mucoprotein complex was identified in the seminal fluid collected in the sperm duct lumen. The sperm ducts showed strong positive expression among negative stained spermatocysts (Figure 3A,B). The cytoplasm of Sertoli cells exhibited strong PAS reaction (Figure 3C). The spermatocytes showed positive PAS/AB reaction (Figure 3D).

**Figure 4:**
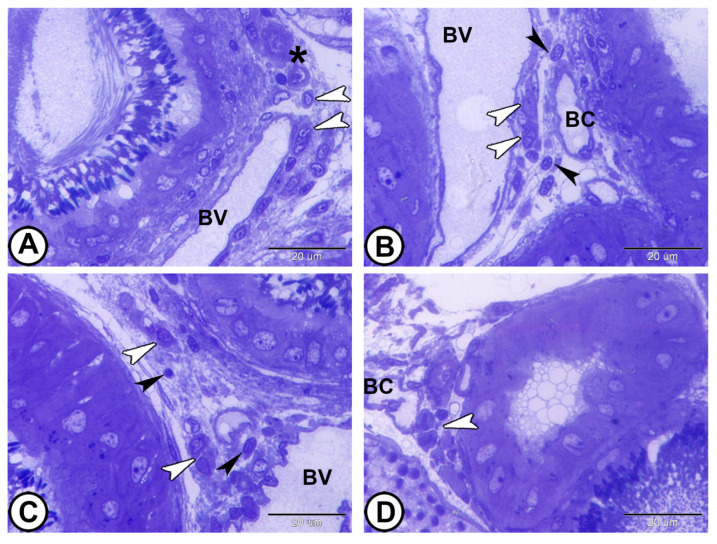
**Semithin sections stained by toluidine blue showing interstitial tissue.** (**A**–**D**) The interstitial tissue contains Leydig cells (white arrowheads), blood vessels (BV), blood capillaries (BC), lymphocytes (black arrowheads), and macrophages (asterisk). (Scale bar: 20 μm in (**A**–**D**)).

The interstitial spaces between the lobules contained Leydig cells, blood vessels, connective tissue, and immune cells, including lymphocytes and macrophages (Figure 4A–D).

**Figure 5:**
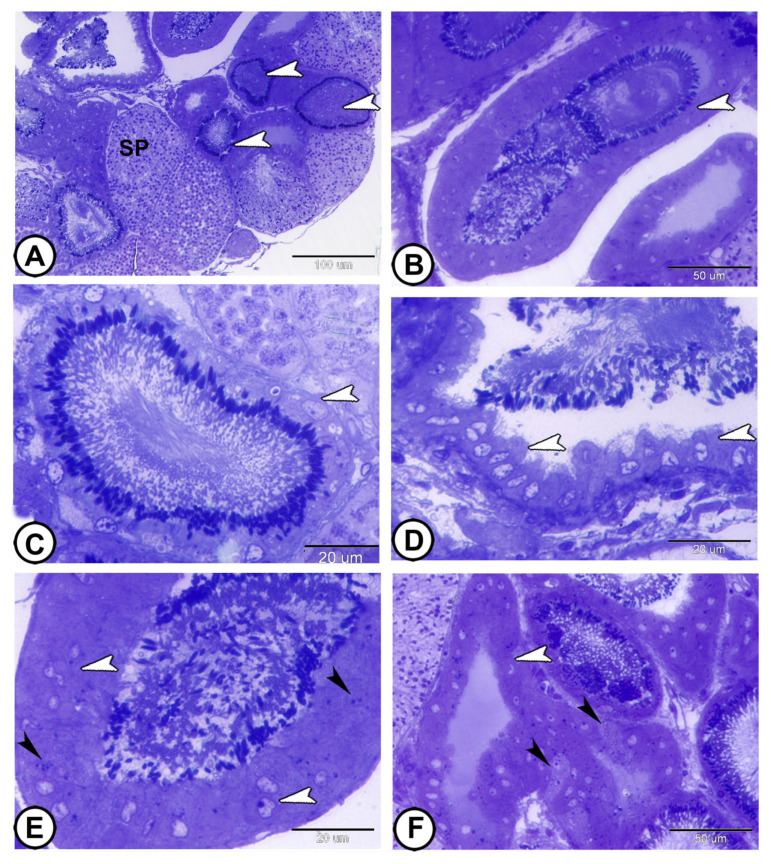
**Semithin sections stained by toluidine blue showing the duct system.** (**A**) The spermatozoa (SP) are released into intralobular lumens, which converge into interlobular ducts or collecting ducts (arrowheads). (**B**,**C**) These initial segments are lined with a simple cuboidal epithelium (arrowheads). (**D**,**E**) The epithelium of the efferent ducts is simple columnar or pseudostratified with microvilli (white arrowheads). The secretory metachromatic granules in the cytoplasm (black arrowheads) are noted. (**F**) The main sperm duct is lined with columnar or pseudostratified (white arrowhead) with secretory cells (black arrowhead). (Scale bars: 100 μm in (**A**); 50 μm in (**B**); 20 μm in (**C**–**E**); and 50 μm in (**F**)).

Developing spermatozoa were discharged into intralobular lumens within the testis, which eventually converged to form collecting ducts or interlobular ducts (Figure 5A). Typically, a basic cuboidal epithelium lines these early segments, and thin connective tissue envelops their walls (Figure 5B,C). Efferent ductules comprise the transitional area between the sperm duct system and the testis. Spermatozoa were directed toward the major sperm duct (vas deferens or ductus deferens) by merging these ducts. The efferent ducts′ epithelium was either pseudostratified with cilia or simple columnar (Figure 5D). The secretory activity was documented in this section (Figure 5E). In this viviparous fish, the primary sperm duct was well-developed and frequently displayed significant morphological specialization. The structure may vary from a narrow, coiled duct to a spacious lumen capable of storing large quantities of spermatozoa. The epithelium is typically columnar to pseudostratified, with secretory cells surrounded by connective tissue lamina propria (Figure 5F). The mean thickness of the epithelium of the efferent duct was 16.20 ± 1.89 µm, while that of the main sperm duct was 22.76 ± 2.36 µm.

### 3.2. Spermatogenic Stage Distribution

**Figure 6:**
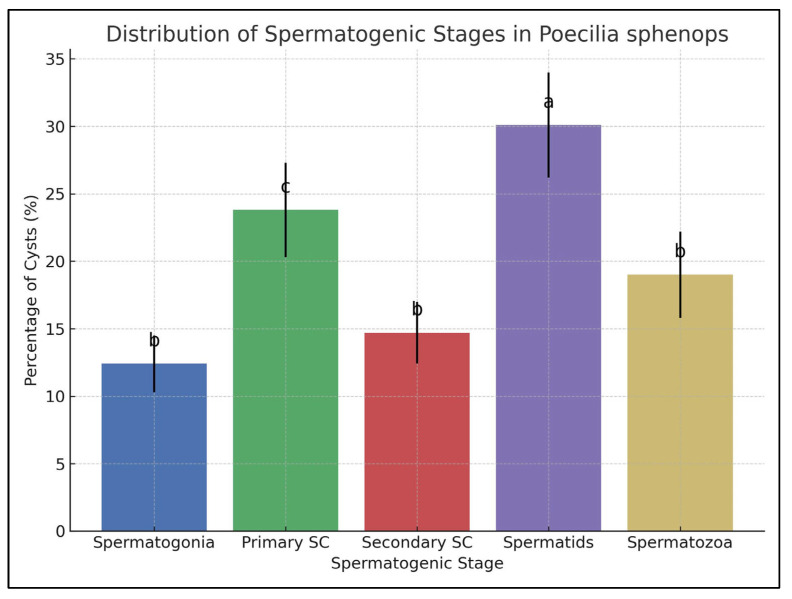
Bar chart showing the mean percentage (± SD) of spermatogenic cysts across 20 specimens. Different superscript letters above the bars (a, b, and c) indicate statistically significant differences among stages (one-way ANOVA, *p* < 0.001; Tukey’s Post Hoc test, *p* < 0.05). **a**: Spermatids were significantly more abundant than all other stages. **b**: Spermatogonia, secondary spermatocytes, and spermatozoa did not differ significantly from each other. **c**: Primary spermatocytes differed significantly from spermatogonia and secondary spermatocytes but not from spermatozoa.

The proportion of spermatogenic cysts showed a predominance of spermatids, followed by primary spermatocytes, spermatozoa, spermatogonia, and secondary spermatocytes. Statistical analysis revealed significant differences among spermatogenic stages (ANOVA, *p* < 0.001). Tukey’s test showed that spermatids were significantly more abundant than all other stages (*p* < 0.05). Primary spermatocytes also differed significantly from spermatogonia and secondary spermatocytes (*p* < 0.05), while no significant differences were detected between spermatogonia, secondary spermatocytes, and spermatozoa (Figure 6). The relative frequency of each stage was consistent across all specimens, reflecting synchronized cystic development typical of restricted lobular testes in poeciliid fish.

### 3.3. Immunohistochemistry

**Figure 7:**
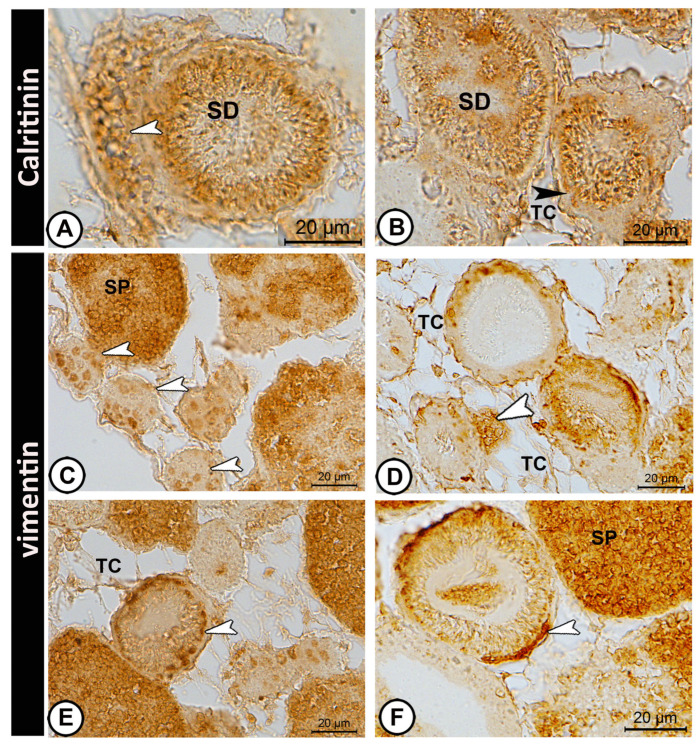
**Immunohistochemistry of calretinin and vimentin.** (**A**,**B**) Calretinin immunoreactivity was localized in interstitial Leydig cells (white arrowhead), epithelium of sperm ducts (SD), and telocytes (TC). Weak expression was found in Sertoli cells (black arrowhead). (**C**) Vimentin was expressed in spermatogonia cysts (arrowhead). (**D**) Interstitial cells showing faint vimentin positivity (arrowhead). (**E**,**F**) Vimentin was expressed in the cytoplasm of Sertoli cells (arrowheads). Telocytes (TC) expressed vimentin in the interstitial tissue. Sperm (SP) expressing vimentin are shown in (**C**,**F**). (Scale bar: 20 μm in (**A**–**F**)).

Calretinin immunoreactivity (Figure 7A,B) was localized in interstitial Leydig cells, the epithelium of sperm ducts, and telocytes. Weak expression was observed in Sertoli cells. No expression was observed in germ cells.

Vimentin was expressed in the spermatogonia cysts (Figure 7C). The interstitial cells also showed faint vimentin positivity (Figure 7D). Vimentin was robustly expressed in the cytoplasm of Sertoli cells, with a filamentous distribution extending along the lobular walls and around developing germ cell cysts. Staining provided a clear structural delineation of the Sertoli cell network (Figure 7E,F). Vimentin was expressed in telocytes in the interstitial tissue (Figure 7D,E). The histological features and immunohistochemical labeling patterns were reproducible and consistent across all examined specimens.

### 3.4. Ultrastructural Characteristics

**Figure 8:**
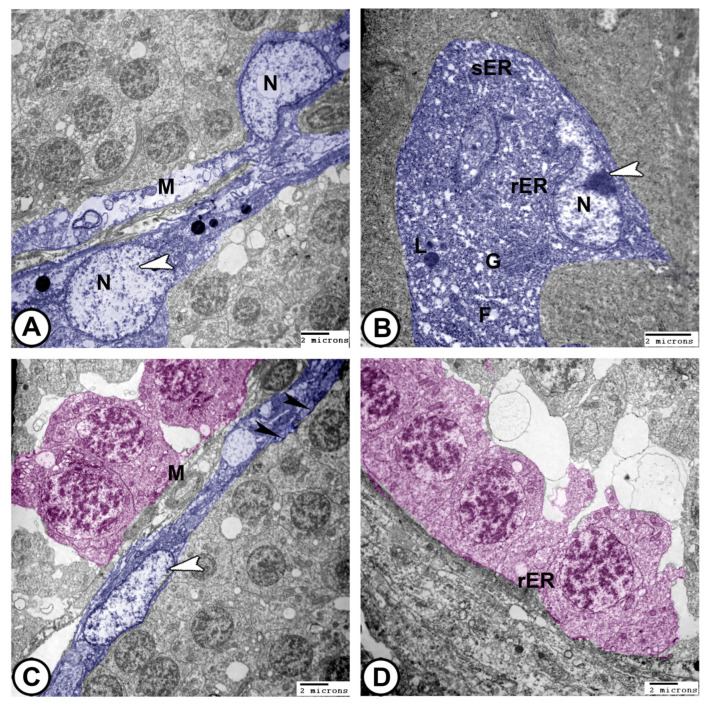
**Digitally colored TEM images of Sertoli cells and spermatogonia.** (**A**,**B**) Sertoli cells (blue) display a large euchromatic nucleus (arrowheads, N). The cytoplasm contains abundant rER, sER, lysosomes (L), mitochondria (M), fat globules (F), and Golgi complexes (G). (**C**,**D**) Spermatogonia (pink) contains mitochondria (M) and sparse rER. Note that Sertoli cells (blue, white arrowhead) are connected to the germ cells and cysts by tight junctional complexes (black arrowheads). (Scale bar: 2 μm in (**A**–**D**)).

**Figure 9:**
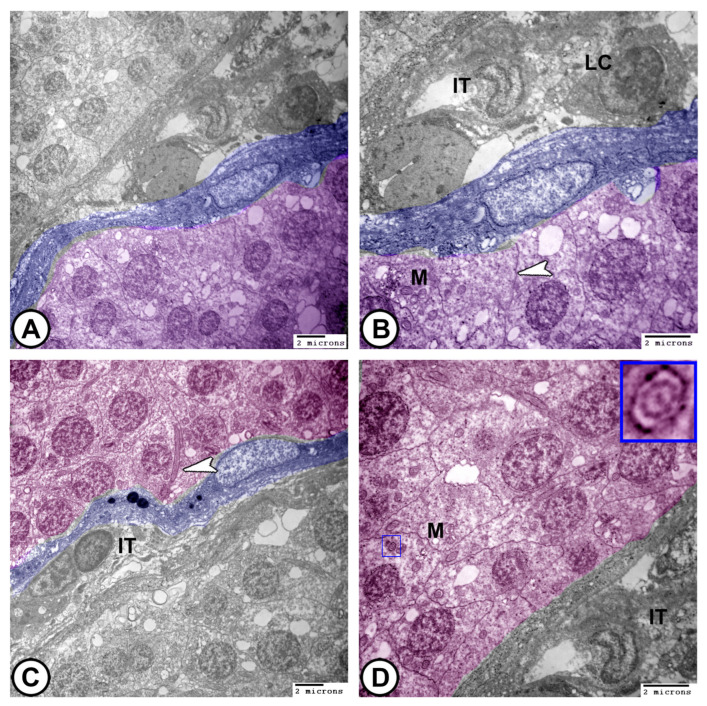
**Digitally colored TEM images of spermatocytes.** (**A**,**B**) Primary spermatocytes (pink) exhibit mitochondria (M), rER, and Golgi apparatus (G). Note the interdigitation junctions between them (arrowhead). (**C**,**D**) Spermatids (violet) are characterized by the presence of developing flagella (arrowhead), characteristic microtubule pattern (boxed areas), and the mitochondria (M). Note that Sertoli cells are presented in blue and interstitial tissues are indicated by IT. (Scale bar: 2 μm in (**A**–**D**)).

**Figure 10:**
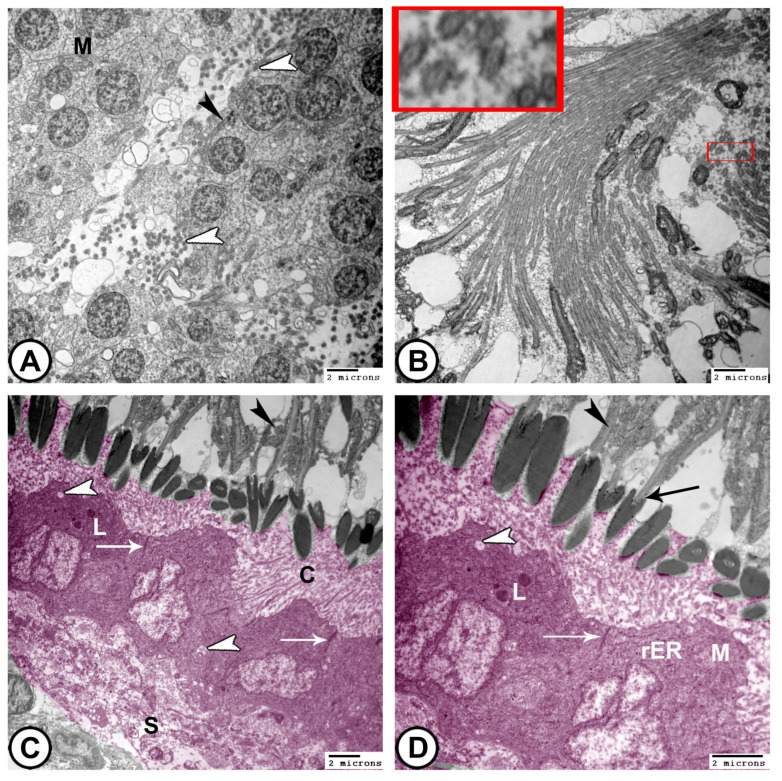
**Digitally colored TEM images of spermatids, spermatozoa, and sperm duct.** (**A**) Spermatids show mitochondria (M), acrosome formation (black arrowhead), and development of the flagellum (white arrowheads). (**B**) Spermatozoa flagella show the typical 9 + 2 axonemal structure, enclosed in the plasma membrane (boxed areas). (**C**,**D**) The epithelium of the sperm duct is covered by short cilia (C). The cytoplasm contains electron-lucent vesicles (white arrowheads), rER, mitochondria (M), and lysosomes (L). Tight junctions and desmosomes are present between adjacent epithelial cells (white arrows). Sertoli cells (S) encircle the sperm duct. The lumen of the sperm duct contains mature spermatozoa with an elongated head (black arrow) and a midpiece with a mitochondrial sheath (black arrowheads). (Scale bar: 2 μm in (**A**–**D**)).

**Figure 11:**
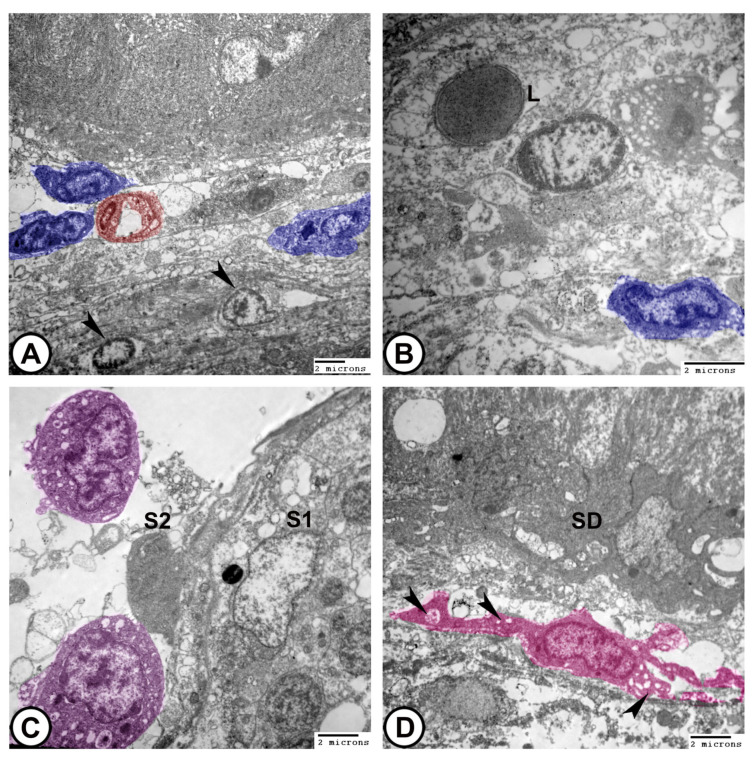
**Digitally colored TEM images of the interstitial tissues.** (**A**,**B**) Leydig cells (blue) around the blood capillaries (red). Note the presence of fibroblasts (arrowheads) and lymphocytes (L). (**C**) Macrophages (violet) appear with large lysosomes, phagosomes, and pseudopodia around spermatocytes. Note the presence of Sertoli cells (S1) around spermatocysts and degenerated Sertoli cells (S2) in association with macrophages. (**D**) Telocytes (pink) with telopodes contain secretory vesicles (arrowheads) around the sperm duct (SD). (Scale bar: 2 μm in (**A**–**D**)).

At the ultrastructural level, transmission electron microscopy (TEM) reveals the following:

(1) **Sertoli cells** were completely encysted, developing spermatogenic cells (Figure 8A). They displayed large euchromatic nuclei with a prominent nucleolus (Figure 8B). The cytoplasm contained abundant rough endoplasmic reticulum (rER), smooth endoplasmic reticulum (SER), lysosomes, mitochondria, Golgi apparatus, and many fat droplets. Sertoli cells were connected to germ cells and cysts by tight junctional complexes (Figure 8A–C).
(2) **Spermatocytes**: Primary spermatocytes exhibited synaptonemal complexes with increased cytoplasmic organelles, including rER, free ribosomes, and mitochondria, during prophase I of meiosis (Figure 8C,D and Figure 9A,B).

(3) **Spermatids**: These typically have a spherical, electron-dense nucleus with unevenly dispersed chromatin was observed in the cells. Numerous mitochondria were arranged in the cytoplasm close to the nucleus, while the endoplasmic reticulum′s cisternae were dispersed throughout the cytoplasm. The flagellum was composed of microtubules arranged in a distinctive “9 + 2” pattern, with nine microtubule doublets surrounding a central pair that were encased in the plasma membrane (Figure 9C,D and Figure 10A).

(4) **Spermatozoa**: They typically have an elongated head, a midpiece with a mitochondrial sheath, and a flagellum (Figure 10B–D).
(5) **The sperm duct** wall was lined with simple columnar epithelium covered by short cilia. The cytoplasm contained numerous electron-lucent vesicles, rER, mitochondria, and lysosomes. Tight junctions (zonula occludens) and desmosomes formed a barrier between adjacent epithelial cells. Sertoli cells were found beneath the epithelium (Figure 10C,D).
(6) **Interstitial tissues** contained Leydig cells around the blood capillaries that were characterized by abundant smooth endoplasmic reticulum, lipid droplets, few lysosomes, and mitochondria, in addition to fibroblasts (Figure 11A,B). Macrophages appeared as irregular-shaped cells with large lysosomes, phagosomes, and pseudopodia (Figure 11C). Telocytes were present in the interstitum and were characterized by a spindle body and telopodes containing secretory vesicles (Figure 11D).

## 4. Discussion

Spermatogenesis in teleost fishes follows a broadly conserved but highly adaptable process, reflecting the species′ reproductive mode [22,23]. In *Poecilia sphenops*, as demonstrated in the present study, spermatogenesis occurs within discrete cysts formed by Sertoli cells that encase germ cells at the same stage of development. This cystic mode of spermatogenesis, which progresses synchronously from spermatogonia to spermatozoa, is characteristic of restricted lobular testes, a structural pattern typical of viviparous teleosts [1]. The progression of germ cell maturation from the periphery toward the lobular lumen, culminating in the release of sperm bundles (spermatozeugmata), aligns with the requirements for internal fertilization [8,24]. In addition to poecilids, all members of the Atheriniformes, Cyprinodontiformes, and Beloniformes groups have been characterized as having this type of testis [25]. Our findings support previous observations in poeciliid species, confirming that this testicular architecture is optimized for controlled, efficient sperm delivery within a viviparous reproductive strategy [26].

While the restricted lobular testis type is commonly shared among viviparous fishes such as *Xiphophorus hellerii*, *Poecilia reticulata*, and *Gambusia affinis* [8,26,27,28], notable differences were observed in the testicular structure of *P. sphenops*. Specifically, the organization of spermatozeugmata within the lobular lumen was more compact and abundant, suggesting species-specific adaptations to sperm storage or delivery [29]. Additionally, the seminiferous lobules of *P. sphenops* appeared to be more tightly packed and showed a higher density of Sertoli cell–germ cell interactions than those reported in some related poecilids [9]. These differences may reflect ecological or behavioral distinctions, such as mating frequency or sperm competition, underscoring the diversity in male reproductive strategies even among closely related viviparous teleosts [29].

Different morphological, behavioral, and physiological adaptations have been developed by viviparous fishes in order to deliver sperm to the female reproductive system. These adaptations include modifications to copulatory organ development, sperm packaging techniques, and mating behaviors [29,30,31]. Molly fish spermatozeugmata are arranged identically to those observed in other poeciliids, with flagella pointing toward the center and sperm heads toward the periphery [8,28,32]. This characteristic is distinct from the sperm arrangement of other inseminating fish species, such as goodeids, which generate spermatozeugmata with irregularly oriented heads [33,34]. Poeciliids and goodeids have independently evolved viviparity, as evidenced by these structural distinctions in spermatozeugmat [33]. Comparative studies have shown that internal fertilization has evolved numerous times within the Atherinomorpha, as evidenced by species belonging to the Anablepidae and Hemiramphidae families, which also exhibit distinct sperm packaging techniques [34]. An additional example relates to sperm transfer techniques; for instance, spermatozoa can be compacted while still in the spermatocyst (Scopaeocharax, Tyttocharax, and Xenurobrycon), or they can be released as free cells into the efferent duct, where they are then compacted by spermatic duct secretions [35]. A modification in the Sertoli–germ cell association alongside an increase in the efferent duct epithelium′s secretory activity contributed to the evolutionary adaptation of spermatozeugmata in poeciliids [30].

The duct system′s structure and intricacy differ between species; the molly fish′s efferent duct system′s simple columnar epithelium has secretory cells that release compounds rich in mucopolysaccharides, which can be visualized by PAS staining. In viviparous teleosts, this type of secretory activity assists in nourishing sperm, encapsulates spermatozeugmata, assists in internal fertilization, and, in certain species, prolongs sperm storage before insemination [9]. It is possible that the mucoproteins in the efferent duct epithelium′s secretions protect spermatozoa from the environment [30].

Molly fish, like the majority of teleost fish, begins spermatogenesis with spermatogonia. Spermatogonia undergo a number of morphological changes during cell division, including an increase in cell number and a decrease in cell size, ultimately resulting in sperm metamorphosis [9]. The primary spermatocytes′ distinctive feature was the presence of protein structures called synaptonemal complexes in the nucleus; these complexes are believed to participate in the homologous chromosome pairing step of meiosis [36]. In the present investigation, we classified spermiogenesis into early and late spermatid stages based on the establishment of the nuclear fossa. A distinctive feature of viviparous teleost spermatozoa is the nuclear fossa, which contains the base of the flagellum and the centriolar complex [37]. The spermatozoa of molly fish exhibit the characteristic elongated shape of poeciliid fishes [25] in contrast to the aquasperm feature of externally fertilizing fish, which possesses spheroidal nuclei [37]. Spermatozoa of inseminating species are thought to have elongated heads as an adaptation that promotes sperm aggregation and the spermatozoa′s passage through the female reproductive canal [38].

Germinal and interstitial compartments are two distinct and separate components of fish testes. Particular cell types with distinctive morphologies were present in each compartment [37]. Germ cells and somatic Sertoli cells were identified in the germinal compartment. The germinal compartment and interstitial tissue were divided by a continuous layer created by the basal surface of the Sertoli cells. Sertoli cells in teleosts differ from those in mammals because their processes form the border of the surrounding individual spermatogonia or spermatocyst and form a germinal epithelium [39]. Therefore, Sertoli cells promote the growth, nourishment, and survival of germ cells as well as control spermatogenesis [1]. We could demonstrate a permeability barrier produced by the tight junction between Sertoli cells and germ cells in molly fish. Similarly to other teleost species, Sertoli cells contained a large number of rER, sER, and lipid droplets in their cytoplasm [40]. Furthermore, we observed many lysosomes in the cytoplasm of the molly fish′s Sertoli cells. Therefore, they play a role in phagocytizing apoptotic germ cells, which are residual bodies discarded by spermatids during spermatogenesis [41]. The interstitial compartment comprises connective tissue and Leydig cells. Fibroblasts, collagen fibers, blood vessels, and immune cells, such as lymphocytes and macrophages, are embedded in the connective tissue. In close proximity to blood vessels, the steroidogenic Leydig cells produce testosterone [34].

The eventual positioning of Sertoli cells following spermatozoa release into the efferent ducts is a debated feature of poecilid spermatogenesis [42]. After spermatozoa are released into the efferent duct, Sertoli cells undergo degeneration similar to that of *P. reticulata* [42]. Conversely, Sertoli cells are integrated into the efferent duct epithelium and acquire secretory capacities in the black molly *Mollienisia latticinna* and *P. Mexicana* [9,27]. Other atherinomorphs, including the goodeids, *Characodon lateralis, Ataenobius toweri,* and *Xenotoca eiseni*, have Sertoli cells that evolve into efferent duct cells following the completion of spermatogenesis [34]. However, in molly fish, we observed that Sertoli cells were degenerated and associated with macrophage cells. We also found Sertoli cells incorporated along the efferent duct. Therefore, within the efferent ducts, Sertoli cells acquire secretory activity. The existence of neutral glycoproteins is indicated by staining their secretions with PAS. Similar findings were found in species belonging to the Cyprinodontidae and Poeciliidae families [7,28].

According to our immunohistochemistry findings, calretinin was expressed in molly fish Leydig cells and weakly expressed in Sertoli cells. The calcium-binding protein calretinin (CALB2) is known to be vital for vertebrate central and peripheral nervous systems [43,44]. In zebrafish (*Danio rerio*), calretinin is extensively expressed in the chemosensory cells and central nervous system [45,46]. The primary role of CALB2 is to prevent excess Ca^2+^ by monitoring and buffering intracellular Ca^2+^ [47]. CALB2 is also essential for necrosis, apoptosis, differentiation, growth, and proliferation of cells [48,49]. The testes and ovaries of mammals express calretinin [50]. However, the role of CALB2 in fish gonads is not widely recognized. Steroid hormone synthesis involves CALB2 [51,52]. The pituitary LH, which is marginally impacted by intracellular calcium levels, controls steroidogenesis and the release of testosterone from Leydig cells [51]. *Paralichthys olivaceus* CALB2 mRNA was substantially expressed in the adult gonads and was more abundant in the testis than in the ovary [52]. Leydig cells displayed positive signals for CALB2, according to the immunohistochemical data, indicating that CALB2 was involved in regulating testosterone production. However, the immunoreactivity of CALB2 was completely missing in spermatogenic cells and weak in Sertoli cells [52]. Our results showed that the spermatozoa in the efferent ducts expressed calretinin. Dressen et al. [53] extended the possible role of calcium-binding proteins in the sperm calcium-signaling cascade, which influences sperm capacitation, motility, and acrosome reaction.

Our immunohistochemical analyses revealed the expression of vimentin in spermatogonia, Sertoli cells, and Leydig cells. Vimentin is a type of intermediate filament that plays crucial functions in the spermatogenic processes, particularly in the differentiation stages of testicular germ cells [54,55]. Furthermore, vimentin is essential for the establishment of cell junctions, regulating the shape and differentiation of spermatogonia, helping to maintain their proper morphology, and mainly attaching growing germ cells to the seminiferous epithelium [56]. Vimentin expression in Sertoli cells has been related to the cells′ mesenchymal origin [57,58]. We show that vimentin is expressed in the efferent duct epithelium, which suggests that these ducts originated from the metamorphosis of Sertoli cells following spermatozoon release [59].

Telocytes are specialized interstitial cells with long, thin projections (telopodes), involved in intercellular signaling and tissue homeostasis [60,61]. Their role in calcium-dependent signaling, secretion, and communication with Leydig cells or blood vessels may explain calretinin expression [62]. Telocytes express vimentin because they are mesenchymal-derived interstitial cells, and vimentin is a hallmark intermediate filament protein in cells of mesenchymal origin s [63].

A limitation of the present study is that it was conducted using a relatively small sample size (20 fish) obtained from a single ornamental supplier during one breeding season. Although this approach reduced genetic and environmental variability and allowed for a focused description of testicular structure, it may limit the generalizability of the findings. Future studies including multiple populations and seasonal replicates would be valuable to confirm the consistency of these observations and to explore possible seasonal or population-level differences in testicular morphology and spermatogenic activity.

## 5. Conclusions

This comprehensive investigation of molly fish (*Poecilia sphenops*) testis offers valuable insights into the structural and functional adaptations associated with viviparity in teleosts. The testis displays a restricted lobular arrangement typical of poeciliids, characterized by synchronously developing germ cell cysts supported by metabolically active Sertoli cells. Ultrastructural analysis revealed distinct stages of spermatogenesis, including synaptonemal complexes in primary spermatocytes and the development of spermatozeugmata, highlighting evolutionary modifications to support internal fertilization. The sperm duct system is well-differentiated, with secretory epithelial cells contributing to sperm conditioning and storage. Calretinin and vimentin immunoreactivity confirmed the somatic identity of Leydig and Sertoli cells, respectively. The presence of vimentin in telocytes and ductal epithelium further suggests a mesenchymal origin and functional integration of these cells in testicular homeostasis. These findings not only enhance the anatomical and cytological characterization of *P. sphenops* but also provide a comparative framework for investigating male reproductive adaptations across teleost lineages.

## Figures and Tables

**Table 1:** The primary and secondary antibodies.

**Primary antibodies**	Calretinin (N-18)	**Supplier**	**Catalog Number**	**Source**	**Dilution**	**Antibody ID**
		Santa Cruz Biotechnology, Santa Cruz, CA, USA	sc-11644	goat	1:100	AB_634545
	Vimentin (RV202)	Thermo Fisher Scientific, Carlsbad, CA, USA	OMA1-06001	mouse	1:100	AB_325529
**Secondary antibodies**	Anti-goat IgG (H+L) HRP conjugate	Thermo Fisher Scientific, Carlsbad, CA, USA	#31402	Rabbit	1:300	AB_228395
	Anti-mouse IgG (H+L) HRP conjugate	Thermo Fisher Scientific, Carlsbad, CA, USA	#31430	Goat	1:300	AB_228307

## Data Availability

The original contributions presented in this study are included in the article/supplementary material. Further inquiries can be directed to the corresponding authors.

## Supplementary Materials

The following supporting information can be downloaded at: https://www.mdpi.com/article/10.3390/vetsci12100930/s1, Figure S1: The Gross morphology of molly fish (*Poecilia sphenops*).; Figure S2: Negative control of calretinin and vimentin immunoreactions in the testis, respectively.; Figure S3: uncolored original TEM images of Figure 8a.; Figure S4: uncolored original TEM images of Figure 9a.; Figure S5: uncolored original TEM images of Figure 10a.; Figure S6: uncolored original TEM images of Figure 11a.
