# Supplementary material for: Unraveling Spermatogenesis in Molly Fish (Poecilia sphenops): An Integrative Study of Testicular Ultrastructure and Immunohistochemistry
Source: Vet Sci. 2025 Sep 24;12(10):930. doi: 10.3390/vetsci12100930 (PMC12567713; doi:10.3390/vetsci12100930)
Supplement: Supplementary file 1 [file vetsci-12-00930-s001.zip › vetsci-3836053-supplementary.pdf]

Unraveling Spermatogenesis in Molly Fish (*Poecilia sphenops*): An Integrative Study of Testicular Ultrastructure and Immunohistochemistry

Doaa M. Mokhtar, Giacomo Zaccone, Marialuisa Aragona, Maria Cristina Guerrera, Rasha Alonizan and Manal T. Hussein

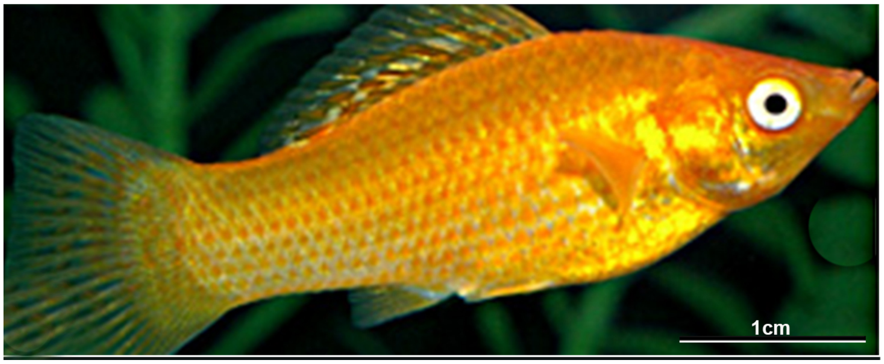

Figure S1: The Gross morphology of molly fish (*Poecilia sphenops*).

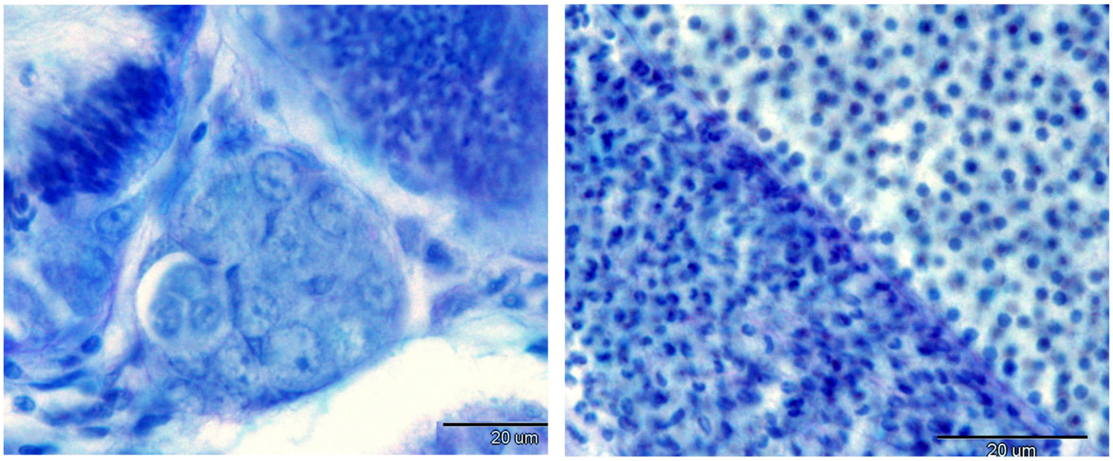

Figure S2: Negative control of calretinin and vimentin immunoreactions in the testis, respectively.

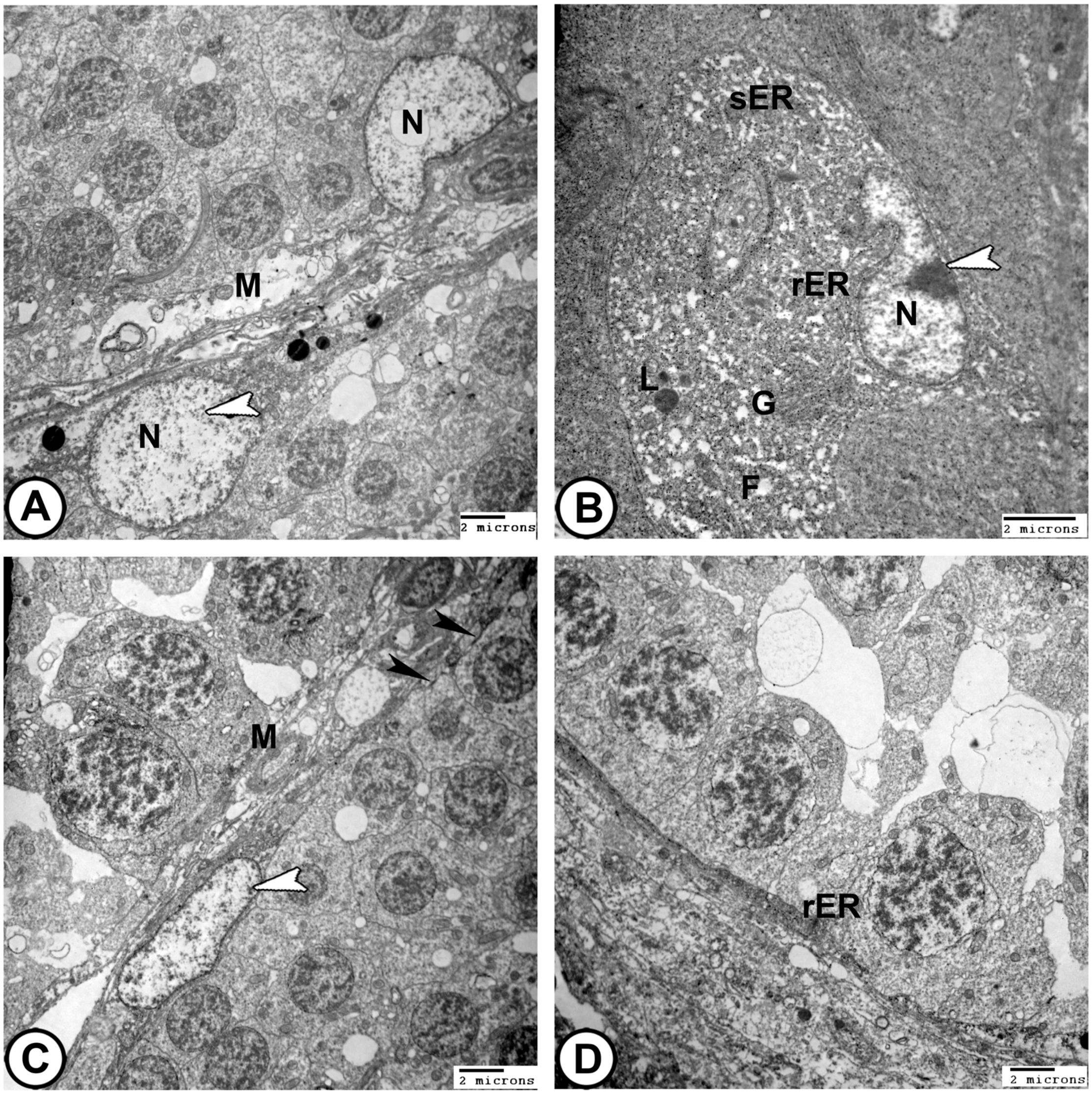

Figure S3 uncolored original TEM images of Figure 8a

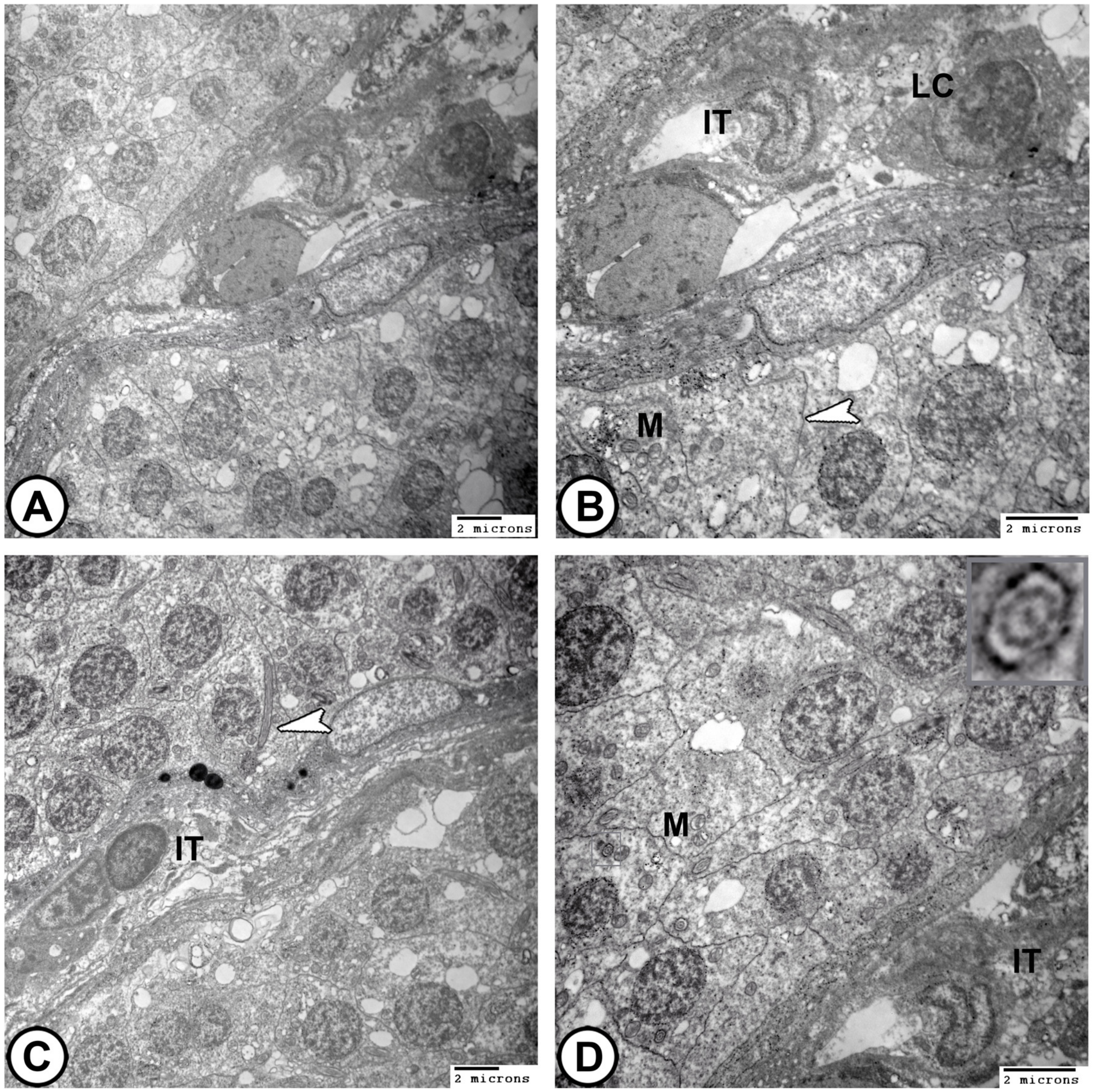

Figure S4 uncolored original TEM images of Figure 9a

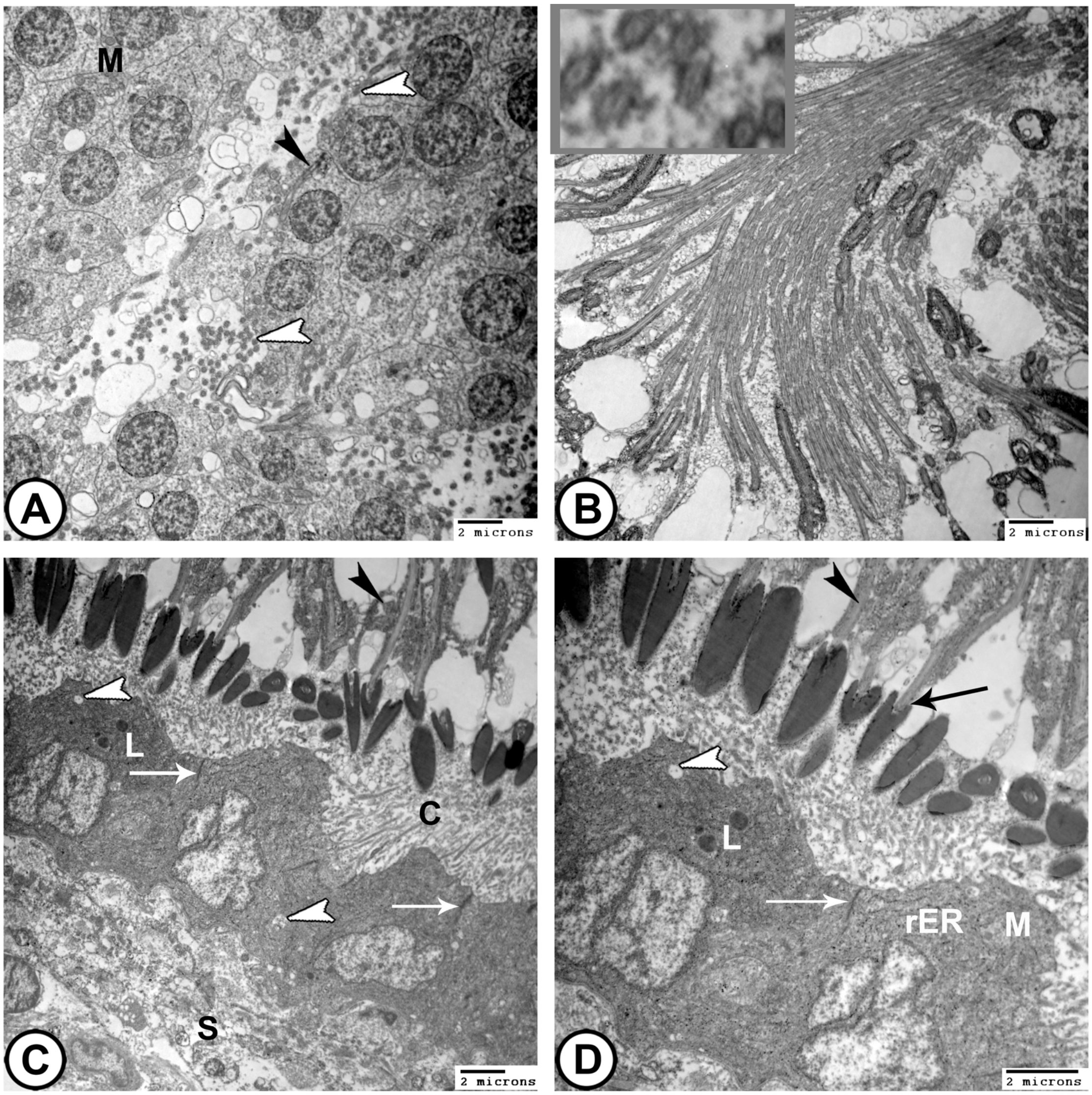

Figure S5 uncolored original TEM images of Figure 10a

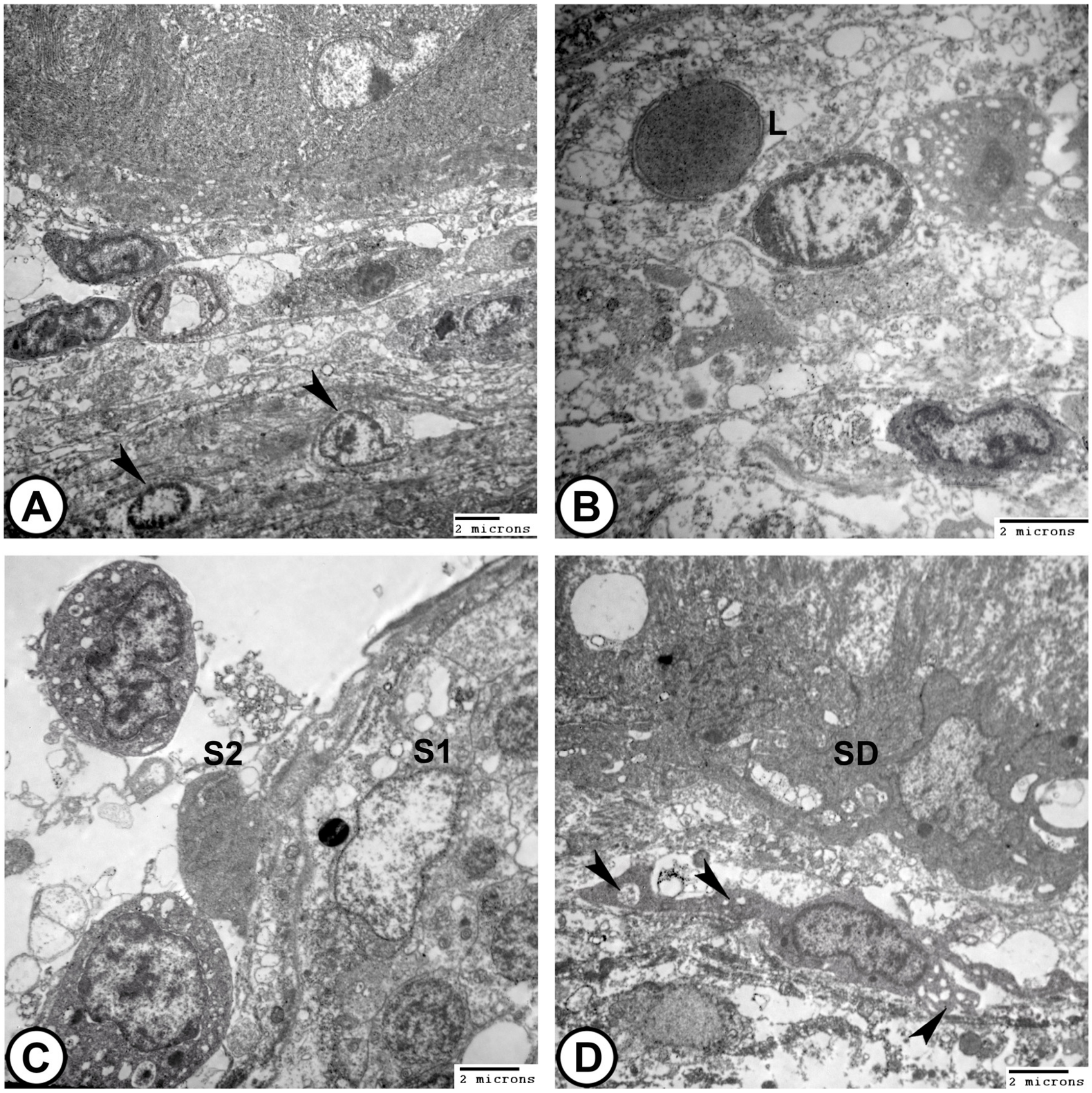

Figure S6 uncolored original TEM images of Figure 11a
